# Modified Interpretations of the Supine Roll Test in Horizontal Canal BPPV Based on Simulations: How the Initial Position of the Debris in the Canal and the Sequence of Testing Affects the Direction of the Nystagmus and the Diagnosis

**DOI:** 10.3389/fneur.2022.881156

**Published:** 2022-05-31

**Authors:** Anita Bhandari, Rajneesh Bhandari, Herman Kingma, Michael Strupp

**Affiliations:** ^1^Vertigo and Ear Clinic, Jaipur, India; ^2^NeuroEquilibrium Diagnostic Systems Pvt Ltd., Jaipur, India; ^3^Faculty of Physics, Tomsk State University, Tomsk, Russia; ^4^Department of Ear Nose Throat, Aalborg University, Aalborg, Denmark; ^5^Department of Otorhinolaryngology, Head and Neck Surgery, Maastricht University Medical Centre, Maastricht, Netherlands; ^6^Department of Neurology, German Center of Vertigo and Balance Disorders, Ludwig Maximilian University, Munich, Germany

**Keywords:** BPPV, horizontal canal, simulation, canalithiasis, supine roll test, maneuvers, nystagmus

## Abstract

**Background and Objectives:**

The aim of this study was to show with three-dimensional simulations how the diagnostic supine roll test (SRT) is affected by the initial position of the debris within the horizontal canal (hc) and study the nystagmus patterns on changing the sequence of testing and its impact on the diagnosis of the side of involvement in hc-BPPV.

**Methods:**

A 3D dynamic simulation model was developed and applied based on reconstructed MRI images and fluid dynamics. Each semicircular canal was linked to the respective extraocular muscles to visualize nystagmus generated on stimulation of the canal.

**Results:**

The simulations of hc-canalithiasis showed that the nystagmus pattern seen with the SRT is changed by the initial position of the otolith debris within the canal and the sequence of testing. The debris changes position during SRT so that sequential steps do not start at the initial position as previously assumed. The sequence of performing the SRT steps from the right or left side influences the nystagmus pattern generated: bilateral direction-changing, bilateral direction-fixed, and unilateral nystagmus can be seen in different test conditions. The SRT itself may even reposition the debris out of the canal.

**Conclusions and Clinical Implications:**

Simulations provide a dynamic tool to study the diagnostic SRT in hc-canalithiasis. Starting the SRT from right or left has a major impact on the test outcome (unlike the Dix-Hallpike maneuver). The findings provide a new interpretation for the results of the SRT. The simulations explain the phenomenon of direction-fixed nystagmus as a logical consequence of starting the SRT with the head turned toward the non-affected side in hc-canalithiasis with debris in the ampullary arm. They also show that unilateral nystagmus seen on SRT indicates canalithiasis of the non-ampullary arm of the side opposite to the side of nystagmus. The generation of bilateral direction-changing, bilateral direction-fixed, and unilateral nystagmus can be the cause of misdiagnoses in terms of the affected side and underlying mechanisms. Finally, a recommendation for a standardized protocol for the sequence of positional tests should be established to ensure uniform interpretation of test results.

## Introduction

Benign paroxysmal positional vertigo (BPPV) is caused by a displacement of otolith debris from the utricle to the semicircular canals. The diagnosis of BPPV is based on the character of nystagmus induced by the various positional tests (1, 2). The Dix-Hallpike test (3) and the side-lying diagnostic Sémont maneuver (4, 5) are used to diagnose posterior canal BPPV, which is associated with torsional, upwardly beating nystagmus. The supine roll test (SRT) is considered useful to diagnose horizontal canal BPPV (hc-BPPV) (6) by eliciting horizontal nystagmus. Other tests like the Bow and Lean test (7) and Upright Roll test (8) have also been described as diagnostic tests for hc-BPPV. Anterior canal BPPV is diagnosed by the supine head-hanging test with the generation of characteristic downwardly beating nystagmus, which may have a torsional component (9, 10).

**Figure 1:**
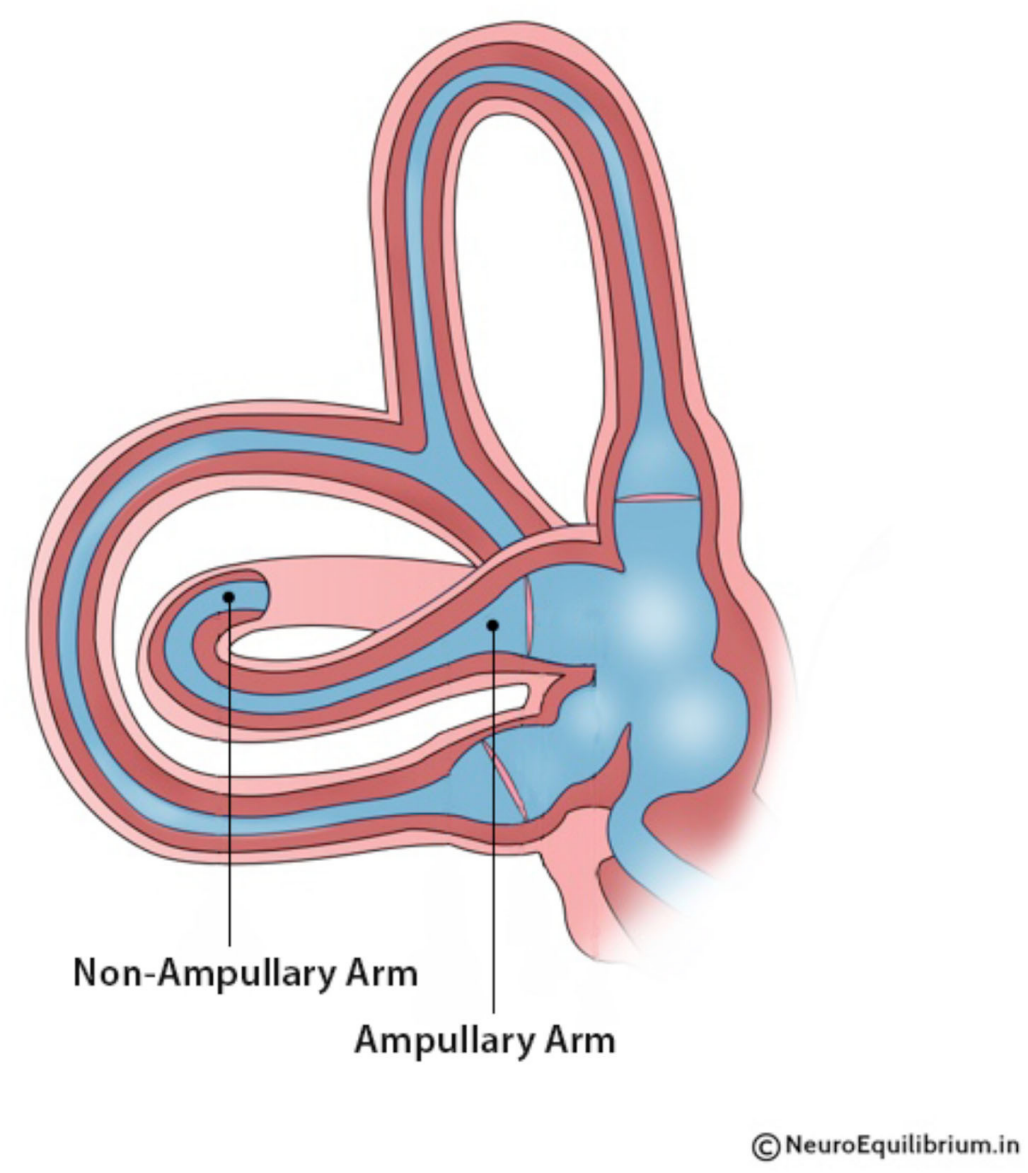
A horizontal canal showing ampullary and non-ampullary arms.

BPPV may occur due to free-floating debris (canalithiasis) (11) or debris adherent to the cupula (cupulolithiasis) (12). The variants of BPPV are also classified based on the position of the debris within the canal (13, 14). For instance, in canalithiasis of the horizontal canal, the debris could be present either in the ampullary or the non-ampullary arm of the canal (15) (Figure 1). Debris in the non-ampullary arm generates geotropic nystagmus, while debris in the ampullary arm generates apogeotropic nystagmus, which can be misinterpreted as hc-cupulolithiasis. The typical clinical picture of hc-canalithiasis has been described as a bidirectional horizontal geotropic nystagmus, which is bi-positional on the right and left sides (16). The present study shows that the latter statement is not always true, as we will show that the direction of the nystagmus depends on whether the affected or the unaffected side is tested first with the diagnostic maneuver.

In this study, we systematically evaluated the different factors that can affect the nystagmus patterns and results of the SRT. Simulations (17) were also used to study how the diagnostic outcome of SRT is affected by the initial position of the debris in the horizontal canal and the side from which the SRT was started. The nystagmus generated in different positions of SRT was visualized with the simulations.

This study (a) demonstrates the effects of the sequence of SRT maneuvers on the direction of the nystagmus, (b) gives an explanation for direction-changing nystagmus vs. direction-fixed nystagmus vs. unilateral nystagmus in different types of hc-canalithiasis, and (c) shows that the assumption that the otolith debris returns to the initial position on turning the head to the opposite side in SRT may be fallacious.

## Methodology

The simulation used a 3D model of the inner ear based on reconstructed images from DICOM files of the temporal bone. The orientation of the canals and the angles between the canals were in accordance with the various reported studies (17, 18). The simulation was created on Unity 3D software. A humanoid was animated within Autodesk Maya with the head linking the semicircular canals to head movement. Otoconial debris in the form of a crystal was put inside the canal using a thin tube (17). By linking the semicircular canals with the respective oculomotor muscles, the nystagmus generated due to canal stimulation was simulated. Ewald's laws have been incorporated in the simulations (19). The time taken for the particle to move at each step of the maneuver was accelerated in all simulations presented here to make it more user-friendly.

### Procedure of Supine Roll Test

- The patient is moved to the supine position with the head inclined forward at an angle of 30° (clinically usually done by placing a pillow under the head).
- The head is turned 90° to the right side and held in this position for 30 s or until the nystagmus subsides.
- The head is turned back to the central position.
- The head is then turned 90° to the left side and held for 30 s or until the nystagmus subsides.
- The head is turned back to the center, and the patient is brought to an upright position.

## Results

Simulations of hc-canalithiasis were used to study the effect of the SRT on the position of otolith debris within the canal. Exemplarily, canalithiasis of the right ear of the ampullary and non-ampullary arms of the horizontal canal was evaluated. In addition, the study compared the effects of starting the SRT from the affected side and the non-affected side.

### Findings of the Simulations of the Four Test Procedures

#### Supine Roll Test for the Right hc-Canalithiasis Non-Ampullary Arm Starting by Rotation to the Right Side

This simulation demonstrates right hc-canalithiasis with the debris in the non-ampullary arm (Supplementary Video 1). The otolith moves toward the ampulla after the head is rotated to the affected side due to gravity. This ampullopetal movement is excitatory. Therefore, geotropic nystagmus beating to the affected right ear is seen. Bringing the head to the center position moves the debris away from the ampulla. When the head is turned to the unaffected left side, the otolith moves toward the utricle. This ampullofugal movement produces an inhibitory stimulus, resulting in geotropic left-beating nystagmus. Thus, direction-changing geotropic nystagmus is seen in hc-canalithiasis when the SRT is started from the affected side.

SRT R—R beating nystagmus.

SRT L—L beating nystagmus.

#### Supine Roll Test for the Right hc-Canalithiasis Non-Ampullary Arm Starting by Rotation to the Left Side

This simulation demonstrates the SRT when starting by turning to the left side in hc-canalithiasis of the non-ampullary arm of the right ear (Supplementary Video 2). When the head is turned to the left, the debris in the right non-ampullary arm moves toward the utricle. This generates left-beating nystagmus. However, the simulation shows that the debris may move out of the canal in this step. Subsequently, no nystagmus is induced when the SRT is performed on the right, as the debris has already been repositioned, and BPPV would be resolved. Thus, unilateral geotropic nystagmus beating to the unaffected side is seen in hc-canalithiasis of the non-ampullary arm when SRT is started from the healthy side. There may be no nystagmus on turning to the affected side.

SRT L—L beating nystagmus.

SRT R—no nystagmus.

#### Supine Roll Test for the Right hc-Canalithiasis Ampullary Arm Starting by Rotation to the Right Side

This simulation demonstrates the performance of SRT, starting by turning to the right side in hc-canalithiasis of the ampullary arm of the right ear (Supplementary Video 3). On turning the head to the right, the debris moves from the ampullary arm to the non-ampullary arm. This results in apogeotropic nystagmus beating to the left. When the head is turned back to the center, the particle remains in the non-ampullary arm. When the head is turned to the left side, the debris moves further through the non-ampullary arm. This generates geotropic left-beating nystagmus. If this position is maintained for longer, it may result in repositioning the debris out of the canal. Thus, starting SRT from the affected side in ampullary arm hc-canalithiasis results in direction-fixed nystagmus with left-beating apogeotropic nystagmus on turning to the affected side and left-beating geotropic nystagmus on turning to the non-affected side. In both positions, the nystagmus is beating to the non-affected side, i.e., direction-fixed nystagmus.

SRT R—L beating nystagmus.

SRT L—L beating nystagmus.

#### Supine Roll Test for the Right hc-Canalithiasis Ampullary Arm Starting by Rotation to the Left Side

This simulation demonstrates SRT, starting by turning to the left side in right hc-canalithiasis with the debris in the ampullary arm (Supplementary Video 4). Turning the head to the left causes little or negligible movement of the debris in the ampullary arm. However, it may convert the effect from canalithiasis to cupulolithiasis with nystagmus generated due to the force effect on the cupula, as seen in Supplementary Video 5. On turning the head to the right, the debris moves away from the ampulla, resulting in left-beating apogeotropic nystagmus.

SRT L—no nystagmus or right-beating prolonged nystagmus

SRT R—L beating nystagmus.

## Discussion

### Supine Roll Diagnostic Test

The SRT is the preferred positional test to diagnose hc-BPPV (2, 24). Precise diagnosis of BPPV, the side affected, and the subtype is critical to successful treatment (25). Determination of the initial location of the debris is based on observation of nystagmus induced by gravity-dependent movements of the otoconia (26). In canalithiasis, the debris may initially be present in the ampullary or non-ampullary arm of the horizontal canal. Debris in the non-ampullary arm generates geotropic nystagmus, which is the more frequent presentation (24) as it is the most dependent region in the upright position. Less commonly, the otoconia may be initially located in the ampullary arm. This position will generate apogeotropic nystagmus on the SRT (6). Clinically, this can lead to the misdiagnosis of cupulolithiasis. The present understanding is that, in the non-ampullary arm involvement, there will be geotropic nystagmus on both sides, and, in ampullary arm involvement, there is apogeotropic nystagmus on both sides (Table 1).

**Table 1:** It presents understanding of the effect of Supine Roll test on horizontal canalithiasis affecting (A) the non-ampullary and (B) ampullary arms (6, 16, 20–23).

	**Start rotating to the right**	**Start rotating to the left**
**A. Debris in the right ear non-ampullary arm**		
SRT right	Right-beating nystagmus	Right-beating nystagmus
SRT left	Left-beating nystagmus	Left-beating nystagmus
**B. Debris in the right ear ampullary arm**		
SRT right	Left-beating nystagmus	Left-beating nystagmus
SRT left	Right-beating nystagmus	Right-beating nystagmus

### 3D-Simulations of SRT Show That the Sequence of Testing Changes Debris Position and Nystagmus Patterns

**Figure 2:**
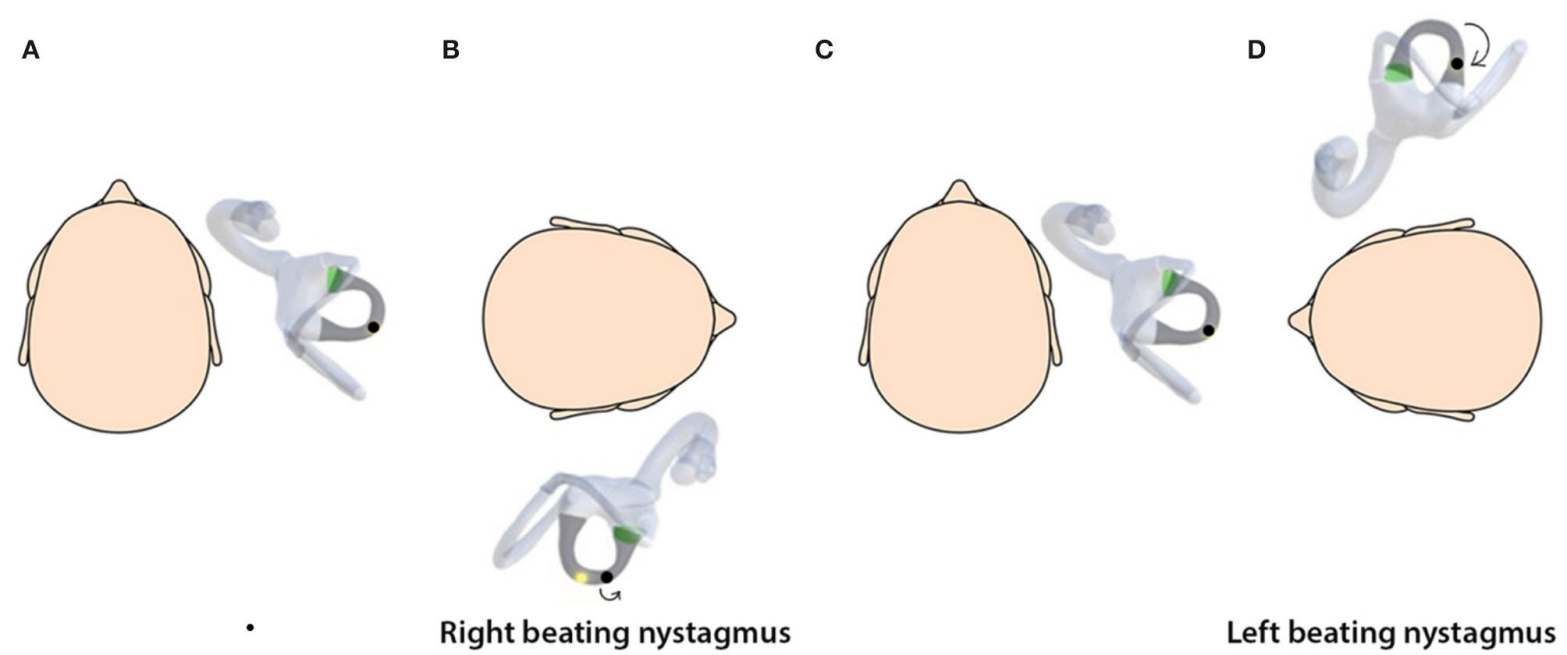
**(A)** Horizontal canalithiasis with debris in the non-ampullary arm. **(B)** Starting SRT by turning to right elicits right-beating nystagmus. **(C)** The head is turned back to the center. **(D)** The head is turned to the left, which elicits left-beating nystagmus. Direction-changing nystagmus is seen.

**Figure 3:**
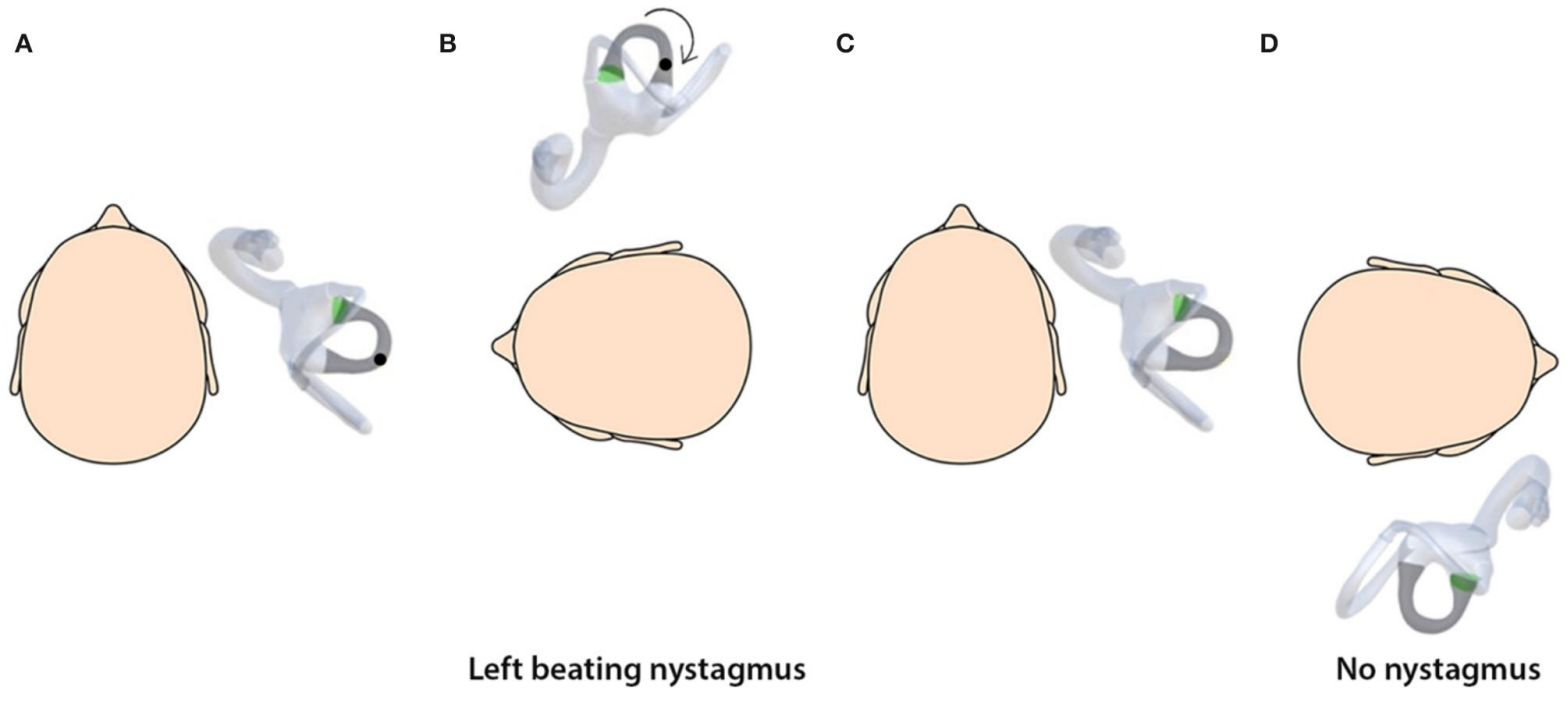
**(A)** Horizontal canalithiasis with debris in the non-ampullary arm. **(B)** Starting SRT by turning to the left elicits left-beating nystagmus. **(C)** The head is turned to the center. **(D)** Turning the head to the right elicits no nystagmus as the debris has been repositioned out of the canal. Thus, unilateral nystagmus is seen on the unaffected side and no nystagmus on the side involved.

**Figure 4:**
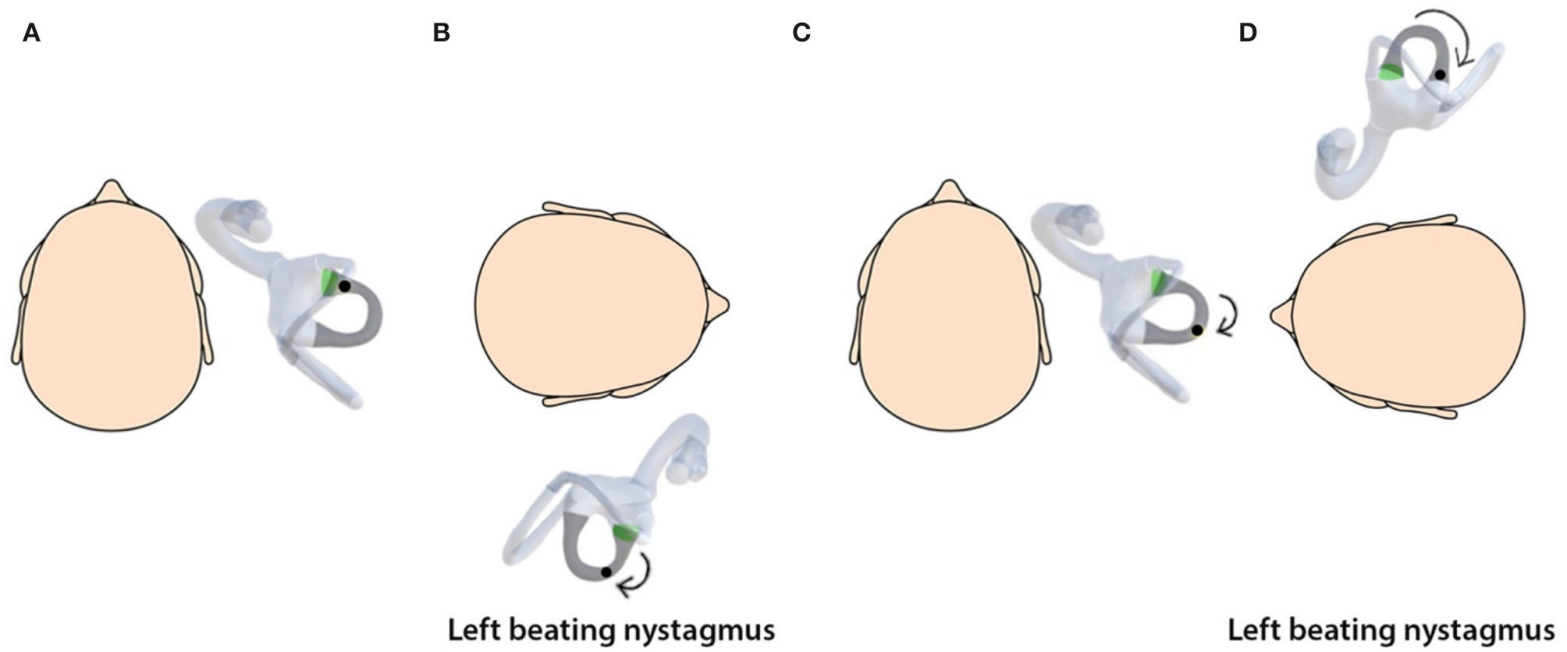
**(A)** Horizontal canalithiasis with debris in the ampullary arm. **(B)** Starting SRT by turning to right elicits left-beating nystagmus. **(C)** The head is turned to the center. **(D)** Turning the head to the left elicits left-beating nystagmus. This is direction-fixed nystagmus.

**Figure 5:**
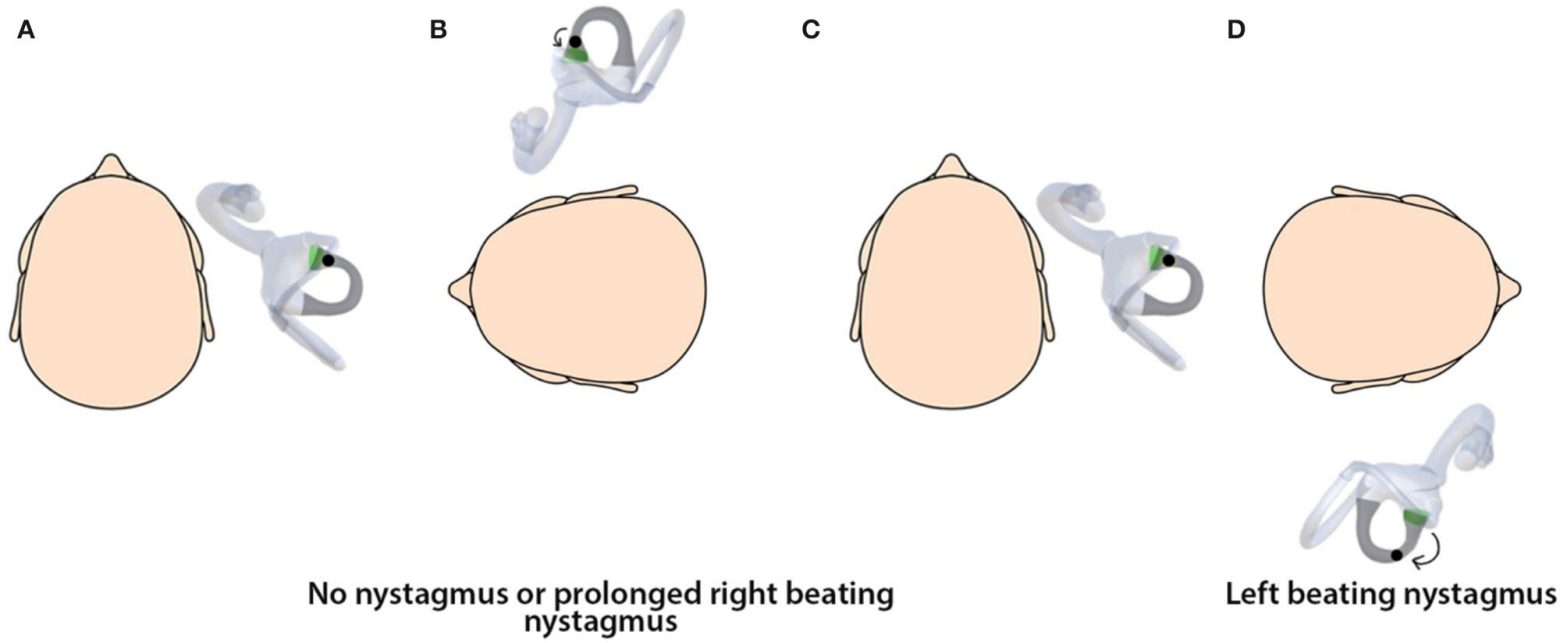
**(A)** Horizontal canalithiasis of the ampullary arm right ear. **(B)** On turning the head to the left in the first step, there is little movement of the debris with no nystagmus elicited, or the debris may exert force on the cupula, leading to generation of prolonged right-beating nystagmus. **(C)** The head is turned to the center. **(D)** On turning the head to the right, there is left-beating nystagmus.

Simulations provide a valuable tool for understanding the three-dimensional spatial movement of the head, semicircular canals, and otoconial debris (18) and the nystagmus induced. The simulations are based on the biophysics of BPPV and depend on basic assumptions regarding the debris size and distribution, the endolymph viscosity, and the canal geometry (15, 27). By linking the semicircular canals with the respective oculomotor muscles, the nystagmus generated on the SRT due to stimulation of canals can also be visualized. An important observation from the simulations is that the otolith debris moves from its original position during the SRT, and the sequence of the diagnostic test affects the clinical findings (Figures 2–5).

Studies have suggested that the particles may not go back to their initial position after the first maneuver such that a second maneuver leads to different particle trajectories, causing smaller cupula displacements (28, 29).

### Nystagmus Patterns: Direction-Changing, Direction-Fixed or Unilateral

The concept of changing otolith position in hc-canalithiasis during the SRT has implications on its results. The simulations show that SRT presents the possibility of the *observer effect*, which refers to the possibility that an act of observation may affect the properties of what is observed (30). Thus, we see that, depending on the side that is tested first, there are variations in nystagmus direction. Intensity of nystagmus is determined by 2 factors:

- Direction of debris movement in the canal—excitatory/inhibitory
- The time for which the gravitational force was applicable on the debris, which is a function of the distance the debris moves

In Figure 2, with the debris in the right non-ampullary arm, on turning to the right, there is an ampullopetal movement, which is excitatory. However, we also see that the distance that the debris moves is much smaller than on turning 90° to the left. In other words, on the right, nystagmus is produced by less debris movement, but it is excitatory. On the left side, there is more debris movement but inhibitory. This could be the reason why the difference between nystagmus intensity on both sides is often, making determination of the side of involvement difficult.

**Figure 6:**
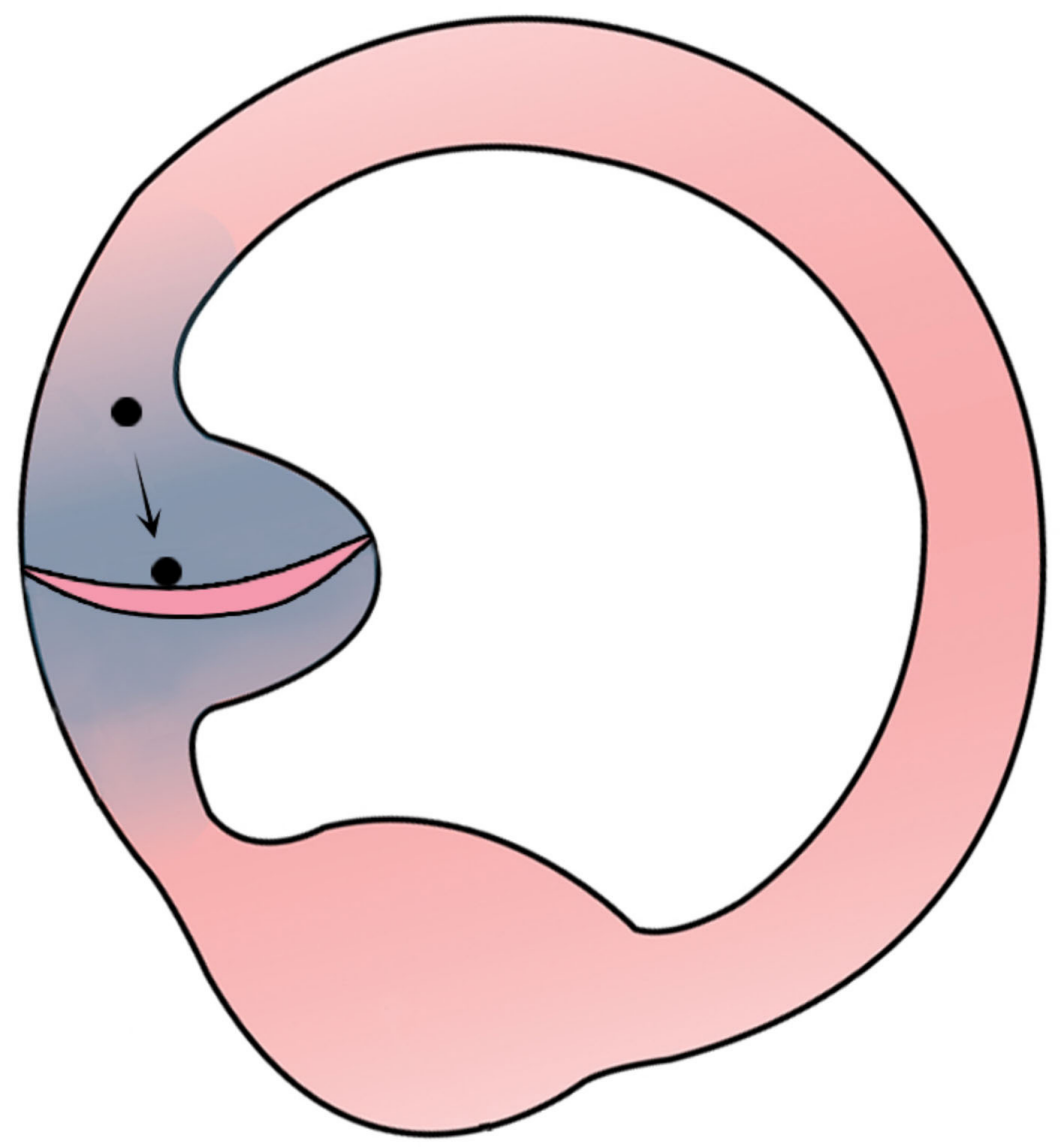
Debris moving from the ampullary arm to exert force on the cupula and produce a cupulolithiasis-like effect.

Due to the changing otolith positions and, consequently, different trajectories, the nystagmus may be bilateral directional-changing (as seen in Supplementary Video 1), bilateral direction-fixed (seen in Supplementary Video 3), or nystagmus on one side only (seen in Supplementary Videos 2, 4) (Table 2; Figures 2–5). Thus, unilateral nystagmus indicates that the affected side is opposite to the side of nystagmus, and the debris may be repositioned by the diagnostic test itself. Supplementary Video 4 shows that, in canalithiasis affecting the ampullary arm, turning the head to the non-affected side causes very little movement of the debris toward the ampulla. As seen in the simulation, if the debris remains in the floating form as canalithiasis, no nystagmus will be generated. However, if the debris exerts force on the cupula, as seen in Supplementary Video 5, it will cause a transition into a cupulolithiasis effect, leading to a very long-lasting apogeotropic nystagmus; i.e., the time constant allows discrimination between canalithiasis and cupulolithiasis (Figure 6).

**Table 2:** Simulation studies of horizontal canalithiasis right ear: (A) Nystagmus patterns of SRT in non-ampullary canalithiasis on starting to the right and left (B) Nystagmus patterns of SRT in ampullary canalithiasis on starting to the right and left.

	**Start rotating to the right**	**Start rotating to the left**
**A. Debris in right ear non-ampullary arm**		
SRT right	Right-beating nystagmus	No nystagmus
SRT left	Left-beating nystagmus	Left-beating nystagmus
**B. Debris in right ear ampullary arm**		
SRT right	Left-beating nystagmus	Left-beating nystagmus
SRT left	Left-beating nystagmus	No nystagmus or right-beating prolonged nystagmus

Some studies have described that, if otoconial debris is located in the ampullary end of the horizontal canal, rotation of the head to the affected side causes the debris to fall away from the ampulla, causing an inhibitory nystagmus that beats toward the unaffected uppermost ear. Rolling onto the opposite side causes debris to return toward the ampulla, triggering a more intense nystagmus, again apogeotropic, beating toward the affected uppermost ear (31, 32). The transition from an apogeotropic nystagmus to geotropic may be due to displacement of otoconia from the ampullary part of the canal to the non-ampullary part. This displacement may be spontaneous or caused by diagnostic maneuvers (6). The conversion of apogeotropic nystagmus to geotropic is called geotropization (33). The transformation into the classical geotropic form suggests the pathophysiological mechanism is canalolithiasis rather than cupulolithiasis, also to be differentiated by the time constant of the nystagmus.

The mechanism of direction-fixed paroxysmal nystagmus in hc-BPPV has been attributed to causes such as canalith jam (13, 26, 32, 33) or multiple otolith masses in different arms of the horizontal canal (34). The transformation of the nystagmus from an apogeotropic to a geotropic form is explained by the migration of the otoconial mass from the ampullary arm of the horizontal canal (20). Supplementary Video 3 shows that ampullary arm hc-canalithiasis, when tested from the affected side, presents with direction-fixed paroxysmal nystagmus, which is directed toward the non-affected side. This is proposed as a logical and straightforward explanation of the mechanism of direction-fixed nystagmus.

### Changing Debris Position During Diagnostic Maneuvers

Many studies have illustrations of the SRT, which show the otolith debris returning to the initial position after turning the head to one side and then back to the center (6, 35, 36) (Table 1). The present study shows that the position of the debris in the horizontal canal is affected by the tests followed (Table 2). Therefore, it may be fallacious to assume that the debris returns to the initial position in the canal on changing head positions. As the particles may be displaced at different steps of the SRT, different testing sequences will alter the nystagmus generated, thus affecting the diagnosis.

### Interpretation of SRT With a Standardized Protocol

By standardizing the sequence of testing during the SRT, it is possible to interpret the initial position of the debris in the canal and the side of involvement. We propose that, in SRT, after going from the sitting to supine position, the head should be turned to the right side first by 90°. The nystagmus is observed. Wait in this position for 30 s or until the nystagmus subsides. Now, turn the head to the center. Wait another 30 s or until the nystagmus subsides, and then turn the head to the left and observe the nystagmus. Finally, the head is turned back to the center.

Our findings from the simulations are seen in Table 3.

**Table 3:** Supine roll test interpretations of hc-canalithiasis on starting the SRT by turning to the right first.

**A. Debris in right ear non-ampullary arm**	
SRT right	Right-beating nystagmus
SRT left	Left-beating nystagmus
**B. Debris in right ear ampullary arm**	
SRT right	Left-beating nystagmus
SRT left	Left-beating nystagmus
**C. Debris in left ear non-ampullary arm**	
SRT right	Right-beating nystagmus
SRT left	No nystagmus
**D. Debris in left ear ampullary arm**	
SRT right	No nystagmus or prolonged left beat
SRT left	Right-beating nystagmus

On the basis of this table, it becomes easier to interpret the findings of SRT. The clinician can decide the side of involvement and initial position of the otolith debris based on the nystagmus pattern. If the nystagmus pattern is as per Tables 3A,B, the maneuver is to be done for the right hc canal, and, if the nystagmus pattern is as per Table 3C or Table 3D, the maneuver is to be carried out for the left hc canal. Another simple way to remember this is to start the SRT by turning to the right side. If you get left beat nystagmus on turning to the left, it means that this is right hc-BPPV. Start the maneuver from the right. Otherwise, start the maneuver from the left.

### Resolution of BPPV During SRT

The simulations also demonstrate why even though horizontal nystagmus was seen on the SRT, no nystagmus may be seen on performing the repositioning maneuver. This can be explained by the fact that the debris has already been repositioned out of the canal, as seen in Supplementary Videos 2, 3.

The success of the treatment of hc-BPPV depends on the correct identification of the pathological side: if the pathological side is not correctly identified, the maneuver may cause the otoconia to move in the wrong direction (6). In addition, the simulations show that different test sequences may generate variable nystagmus patterns. Therefore, it is crucial for the clinician to follow the nystagmus pattern to ascertain the position of the debris within the canal to perform the correct treatment maneuver.

## Limitations

Our study is based on the orientation of the semicircular canals obtained from the reconstructed MRI images. We are aware that our simulations and underlying physics model cannot precisely represent the *in vivo* movement of the otoconia in the semicircular canals of each patient because there are many variables among patients. The simulations we have used do not take into account the impact of different debris sizes and the possibility that the debris can be located in different parts of the canal at the same time. There are several unknown variables, and visualizing the otolith movement for each patient is beyond the scope of our study. In this study, we have demonstrated only simulations of canalithiasis and excluded cupulolithiasis. We encourage clinical validation of our theoretical results, i.e., randomized controlled clinical trials directly comparing the findings of the various tests discussed here.

## Conclusions and Clinical Implications

The simulations provide a helpful tool to dynamically understand the orientation of the head, horizontal canal, and otolith debris in different positions in three-dimensional space, along with visualization of the nystagmus generated. The paper provides a new interpretation of the findings of SRT. The study shows how the sequence of the diagnostic tests affects the otolith position and impacts test results. The simulation model can explain varying nystagmus patterns seen in hc-canalithiasis, such bilateral direction-changing nystagmus, bilateral direction-fixed nystagmus, and nystagmus on only one side. The simulations explain the phenomenon of direction-fixed nystagmus as a logical consequence of starting the SRT with the head turned toward the non-affected side in hc-canalithiasis with debris in the ampullary arm.

Unilateral nystagmus seen on SRT indicates canalithiasis of the non-ampullary arm of the side opposite to the side of nystagmus. Identifying these different nystagmus patterns will help the clinician determine the side of involvement and location of the debris within the canal. It is recommended that a standard sequence for positional testing be followed globally to ensure uniformity in test conditions and interpretation of results.

## Data Availability Statement

The original contributions presented in the study are included in the article/Supplementary Materials, further inquiries can be directed to the corresponding author.

## Ethics Statement

Ethical review and approval was not required for the study on human participants in accordance with the local legislation and institutional requirements. Written informed consent to participate in this study was provided by the patients.

## Author Contributions

AB and RB: conceptualization and development of 3D simulation. AB: formulated study design and writing the manuscript. RB and MS: contribution to study design. AB, RB, HK, and MS: interpretation of data. MS: conception of the study, drafting, and editing of the manuscript for intellectual content. HK: development of simulation models, improvement of debris movement, visualization, and optimization of manuscript. All authors reviewed and approved the manuscript.

## Conflict of Interest

MS is the joint chief editor of the Journal of Neurology, editor in chief of Frontiers of Neuro-otology and section editor of F1000. He has received speaker's honoraria from Abbott, Auris Medical, Biogen, Eisai, Grünenthal, GSK, Henning Pharma, Interacoustics, J&J, MSD, Otometrics, Pierre-Fabre, TEVA, UCB, and Viatris. He is a shareholder and investor of IntraBio. He distributes “M-glasses” and “Positional vertigo App.” He acts as a consultant for Abbott, AurisMedical, Heel, IntraBio, and Sensorion. RB is the director of NeuroEquilibrium Diagnostic Systems Private Limited, India. The remaining authors declare that the research was conducted in the absence of any commercial or financial relationships that could be construed as a potential conflict of interest.

## Publisher's Note

All claims expressed in this article are solely those of the authors and do not necessarily represent those of their affiliated organizations, or those of the publisher, the editors and the reviewers. Any product that may be evaluated in this article, or claim that may be made by its manufacturer, is not guaranteed or endorsed by the publisher.

## Abbreviations

BPPV: Benign paroxysmal positional vertigo
hc: horizontal canal
ca: canalithiasis
SRT: Supine roll test.
